# Inflammatory response to elective percutaneous coronary intervention is related to myocardial injury

**DOI:** 10.31744/einstein_journal/2025AO1582

**Published:** 2025-09-11

**Authors:** Priscilla Teixeira Céo Frisso, Henrique Trombini Pinesi, Fernando Ramos de Mattos, Antonio Eduardo Pereira Pesaro, Fabiana Hanna Rached, James Andrew de Lemos, Marcelo Franken, Adriano Caixeta, Antonio Carlos Palandri Chagas, Pedro Alves Lemos, Carlos Vicente Serrano

**Affiliations:** 1 Universidade de São Paulo Faculdade de Medicina Hospital das Clínicas São Paulo SP Brazil Instituto do Coração, Hospital das Clínicas, Faculdade de Medicina, Universidade de São Paulo, São Paulo, SP, Brazil.; 2 Hospital Israelita Albert Einstein São Paulo SP Brazil Hospital Israelita Albert Einstein, São Paulo, SP, Brazil.; 3 The University of Texas Southwestern Medical Center - Cardiology Dallas Texas United States The University of Texas Southwestern Medical Center - Cardiology - Dallas, Texas, United States.; 4 Universidade Federal de São Paulo Escola Paulista de Medicina São Paulo SP Brazil Escola Paulista de Medicina, Universidade Federal de São Paulo, São Paulo, SP, Brazil.; 5 Centro Universitário FMABC Santo André SP Brazil Centro Universitário FMABC, Santo André, SP, Brazil.

**Keywords:** Percutaneous coronary intervention, Acute coronary syndrome, Myocardial injury, Neutrophis, Systemic inflammatory response, Prognosis, Inflammation, Biomarkers

## Abstract

**Introduction:**

In acute coronary syndrome, the release of inflammatory and myocardial injury biomarkers after percutaneous coronary intervention is associated with poor prognosis. However, little is known about the association between these biomarkers in patients with stable disease.

**Objective:**

We aimed to analyze the association between the systemic inflammatory response and myocardial injury after percutaneous coronary intervention in patients with chronic coronary syndrome.

**Methods:**

In this retrospective observational study, we collected blood sample data from 329 patients with chronic coronary syndrome before and 48 hours after successful percutaneous coronary intervention. Inflammatory biomarkers, including high-sensitivity C-reactive protein (CRP), white blood cells and subtypes, platelets, mean platelet volume, neutrophil-to-lymphocyte ratio, and platelet-to-lymphocyte ratio, were determined. High-sensitivity cardiac troponin I (hs-cTnI) was measured within 48 hours after percutaneous coronary intervention. Procedural myocardial injury was defined as an increase in hs-cTnI level without any new electrocardiographic changes or flow-limiting complications.

**Results:**

Percutaneous coronary intervention induced a systemic inflammatory response, with elevated levels of inflammatory biomarkers. Myocardial injury was detected in 66.7% of the patients. Spearman's rank-order correlation revealed an association between hs-cTnI and increased inflammatory biomarker levels: CRP 0.570 (p<0.001), neutrophil-to-lymphocyte ratio 0.190 (p<0.001), mean platelet volume 0.182 (p<0.001), platelet-to-lymphocyte ratio 0.180 (p<0.001), white blood cells 0.166 (p<0.001), neutrophils 0.145 (p<0.001), eosinophils 0.142 (p<0.001), lymphocytes −0.130 (p<0.001), monocytes 0.031 (p=0.344), and platelets −0.009 (p=0.778).

**Conclusion:**

Percutaneous coronary intervention in patients with chronic coronary syndrome induces a systemic inflammatory reaction, which is associated with myocardial injury.

## Highlights

■Previous studies have correlated inflammation with complications after percutaneous coronary intervention.

■In patients with stable coronary disease, percutaneous coronary intervention leads to elevated inflammatory biomarker levels.

■The inflammatory response is significantly correlated with myocardial injury.

## In Brief

This retrospective cohort study was designed to evaluate the effect of percutaneous coronary intervention with stent implantation on the inflammatory response in patients with stable coronary disease. Percutaneous coronary intervention led to elevated levels of inflammatory biomarkers. There was an association between the elevated levels of inflammatory biomarkers and myocardial injury in this population.

## Graphical abstract

**Figure:**
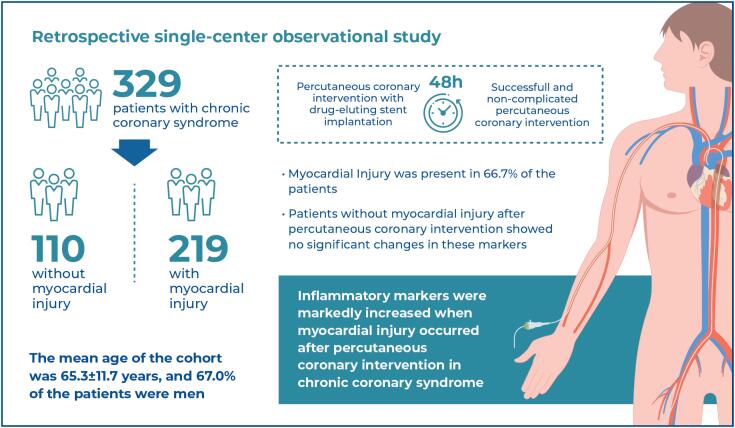

## INTRODUCTION

The treatment of chronic coronary syndrome (CCS) focuses on improving outcomes and relieving symptoms. The guidelines for obstructive coronary artery disease recommend revascularization in patients with limiting and refractory symptoms. In this context, percutaneous coronary intervention (PCI) is often performed, particularly in patients with lower clinical and angiographic risks.^(1)^

However, patients undergoing PCI are at risk of restenosis. Studies have shown that coronary artery stents stimulate an early inflammatory response concentrated at the stent struts within the first three days.^(2)^ This response is driven by disruption of the endothelial layer, release of inflammatory mediators, and activation of leukocytes, such as neutrophils, monocytes, and lymphocytes.^(3)^

Proinflammatory mediators released during arterial wall injury play a central role in restenosis.^(4)^ Among these mediators, high-sensitivity C-reactive protein (hs-CRP), and more recently, hematological indices, have received substantial attention.^(5)^ These indices, also known as leukocyte-derived parameters, such as the neutrophil-to-lymphocyte ratio (NLR) and platelet-to-lymphocyte ratio (PLR), have recently been shown to be highly sensitive biomarkers of inflammatory disorders. These hematological indices have also been widely used to determine the severity of inflammation and as predictors of poor outcomes in patients with cardiovascular diseases. Importantly, these indices do not have a direct pathophysiological role in procedure-related inflammation, unlike reactive oxygen intermediates and inflammatory cytokines.^(6)^

An increase in high-sensitivity cardiac troponin I (hs-cTnI) levels following effective PCI is common and is defined as myocardial injury related to this procedure.^(7)^ Postprocedural elevations of troponin and/or creatine kinase muscle-band levels occur in 5-50% of patients undergoing successful PCI. However, the clinical impact remains unclear.^(8)^ The introduction of the hs-cTnI assay into clinical practice, which can distinguish small elevations of this biomarker, makes Tn increments following PCI even more controversial.^(9)^ In the "Fourth Universal Definition of Myocardial Infarction",^(10)^ PCI-related MI (type 4a) was subjectively denoted by an increase in Tn values of more than five times the 99th percentile upper reference limit in patients with normal baseline levels. In addition, one of the following factors is required to define type 4a MI: symptoms of myocardial ischemia, novel ischemic electrocardiographic alterations, angiographic findings reliable with a procedural drawback, loss of myocardium, or novel regional wall motion defects demonstrated by imaging analyses. A significant increase in Tn levels without these elements is arbitrarily defined as procedure-related myocardial injury.

Taking this into account, this study was conducted based on the hypothesis that procedure-related myocardial injury in patients with CCS is associated with an enhanced inflammatory response.

## OBJECTIVE

This study aimed to analyze the correlation between myocardial injury and inflammation following percutaneous coronary intervention in patients with chronic coronary syndrome. hs-CRP and hematological indices were used to assess inflammation. The approach proposed was to compare patients with chronic coronary syndrome with and without myocardial injury according to the magnitude of the inflammatory response after elective percutaneous coronary intervention.

## METHODS

### Study population

This retrospective study included 329 patients with CCS who underwent elective PCI for revascularization at a single center. All patients had baseline hs-cTnI levels within reference ranges. Indications for PCI were independent of this study, as approved by the Ethics and Research Committee.

The diagnosis of CCS was based on the occurrence of chest pain for more than two months with a positive treadmill test and/or triggered by demanding exertions, at least one-vessel disease (70-90% stenosis), and standard left ventricular function. The presence of these features characterized a patients as being at a low risk.^(11)^

The exclusion criteria were PCI-related MI (type 4a), acute coronary syndrome within 3 months before PCI, percutaneous or surgical revascularization of less than 6 months, hemodynamic impairment, left ventricular dysfunction (defined as ejection fraction <50%), urgent revascularization, acute or chronic infection or inflammatory state, and the presence of stent restenosis.

This study was approved by the Research Ethics Committee of *Hospital Israelita Albert Einstein*, CAAE: 54065516.1.0000.0071; # 1,463,667.

### PCI procedure and adjunctive therapy

Elective PCI with drug-eluting stent implantation and medical treatment was performed in accordance with the Brazilian Society of Cardiology guidelines.^(12)^At the time of the procedure, all patients were receiving statin and dual antiplatelet therapy. The customary expected interval between the diagnostic angiography and planned PCI was 7 days. Throughout the stent intervention, all patients received 100U/kgunfractionated heparin intravenously and were monitored for activated clotting time thereafter.

### Biomarker measurements

Measurement of hs-cTnI and other hematological biomarkers, such as LDL cholesterol, creatinine, and complete blood count, was performed using institutional laboratory methods. hs-cTnI was measured with a two-site chemiluminescent enzyme immunometric assay (IMMULITE^®^ 1000 Chemiluminescent Technology, Siemens Healthcare Diagnostics, Los Angeles, CA) 48 hours after PCI. However, if the troponin levels increased at this time point, serial measurements were performed until the elevations resolved. Procedural myocardial injury was defined as post-PCI hs-cTnI values greater than 170 pg/ml without new ECG changes or flow-limiting complications.

hs-CRP was measured using a high-sensitivity nephelometric method (BN II, Dade Behring Inc., Newark, DE), which determines the agglutination of particles by quantifying the scattered light (detection limit >0.175 µg/dl). The intra-assay flexibility for the lower range was <0.165 µg/dl. hs-CRP values above 3.0µg/dl were considered as elevated. Leukocytes and subtypes, platelets, mean platelet volume (MPV), NLR, and PLR were also determined before and 48 hours after PCI.

### Study protocol

In this investigation, we used sociodemographic, clinical, laboratory, and angioplasty-related data extracted from medical records. Laboratory data were obtained from peripheral blood samples collected immediately before and after successful uncomplicated PCI. Ethylenediaminetetraacetic acid was used as the anticoagulant. Blood samples were used within 2.5 hours of collection, and patients were grouped according to the presence of myocardial injury after PCI.

### Statistical methods

The number of participants required for the study was based on previous reports. These reports indicated that 247 patients with CCS were needed to detect a 20% increase in hs-CRP based on a power of 0.90 and an alpha value of 0.05. Consequently, we enrolled patients with and without myocardial injury until we had a sufficient number of individuals in each group.

Medians (minimum, maximum), means (± standard deviation), and percentages were used. Baseline data of patients with and without myocardial injury were analyzed using Student's *t*-test for continuous variables or Fisher's exact test for categorical variables. The preprocedural levels of all biomarkers were determined using the Wilcoxon rank-sum test. Spearman's test was used to measure the strength, direction, and order of the correlation between increased hs-cTnI and IBM levels. The SAS software was used for statistical analysis.^(13)^ Comparisons between baseline and each time point were performed for each patient, and the mean comparisons were related to zero using repeated-measures analysis of variance of the log-transformed data. All statistical analyses were defined as significant when p<0.05.

## RESULTS

### Baseline characteristics

The baseline characteristics, including demographic, laboratory, clinical, angiographic, and medication descriptions of patients who progressed without (n=110) and with (n=219) myocardial injury after PCI are displayed in table 1. Most baseline characteristics were comparable between the two groups. The mean age of the cohort was 65.3 ± 11.7 years, and 67.0% of the patients were men. Patients with a significant increase in hs-cTnI levels had more unfavorable clinical characteristics: they were older, had higher baseline blood pressure and LDL levels, had a history of MI and revascularization, and used fewer beta-blockers. There were no statistically significant differences in the use of antiplatelet agents or statins. In addition, the left anterior descending artery was the most commonly treated vessel in both groups of patients, being more prevalent in those who developed myocardial injury.

**Table 1:** Baseline characteristics of the study population according to the presence or absence of myocardial injury after elective percutaneous coronary intervention

		Without myocardial injury (n=110)	With myocardial injury (n=219)	p value
Demographic, laboratory, and clinical characteristics				
	Age (years)	64.9 ± 11.0	65.3 ± 9.0	0.01
	Men (%)	66	67	NS
	Hypertension (%)	24	27	0.047
	Post-PCI hs-cTnI (pg/ml)	123.1 ± 45.0	199.9 ± 23.3	<0.001
	LDL-cholesterol (mg/dl)	72 ± 18	77 ± 32	0.001
	Creatinine (mg/dl)	1.1 ± 0.1	1.2 ± 0.2	NS
	Previous myocardial infarction (%)	8	10	0.009
	History of PCI/CABG (%)	12	14	0.008
Treated vessel with PCI				
	LADA (%)	45.0	59.1	0.007
	LC×A (%)	8.1	11.2	0.01
	RCA (%)	31.0	23.1	0.009
Most common hospital medications used				
	Aspirin (%)	98	99	NS
	P2Y12 inhibitor (%)	100	100	NS
	Statin (%)	100	100	NS
	Beta-blocker (%)	35	39	0.001
	ACE inhibitors/ARB (%)	25	22	0.003

Data are expressed in mean ± standard deviation.

hs-cTnI: high-sensitivity cardiac troponin I; PCI: percutaneous coronary intervention; CABG: coronary artery bypass graft surgery; LADA: left anterior descending artery; LCxA: left circumflex artery; RCA: right coronary artery; ACE: angiotensin-converting enzyme; ARB: angiotensin receptor blocker; NS: not significant.

Overall, new Q-wave MI within 48 hours of PCI was observed in 3 patients, while 5 patients had no-reflow (TIMI flow grade <2).

### Procedural levels of IBM

As shown in table 2, all patients had normal IBM levels before and immediately after PCI. However, after the procedure, a sharp increase in IBM levels was observed in all the patients.

**Table 2:** Inflammatory biomarkers before and after percutaneous coronary intervention in stable ischemic heart disease patients

Biomarkers	Before PCI n=329	After PCI n=329	RV	p value
WBC (× 10^3^/μl)	9.5	10.4	3.5 - 10.5	<0.001
Neutrophils (%)	68.1	69.1	48 - 70	<0.001
Eosinophils (%)	6.0	6.9	1 - 7	<0.001
Lymphocytes (%)	35.2	34.5	34 - 50	<0.001
Monocytes (%)	20.0	21.0	20 - 27	<0.001
Platelets (×10^3^/μl)	194.3	195.2	150 - 450	<0.001
MPV (fl)	12.0	12.9	6.5 - 15.0	<0.001
hs-CRP (µg/dl)	6.4	9.0	0.0 - 0.3	<0.001
NLR	2.1	2.2		<0.001
PLR	5.8	6.0		<0.001

Student's *t* test was applied.

RV: reference value; WBC: white blood cell; MPV: mean platelet volume; hs-CRP: high-sensitivity C-reactive protein; NLR: neutrophil-lymphocyte ratio; PLR: platelet-lymphocyte ratio; PCI: percutaneous coronary intervention.

**Figure 1:**
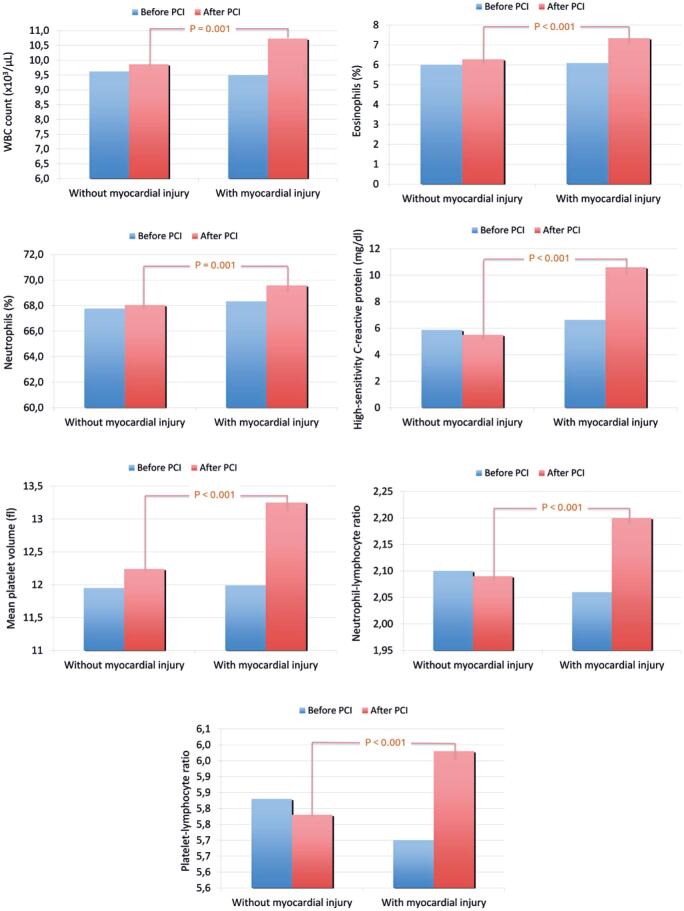
Inflammatory biomarkers, such as white blood cell count, eosinophils, neutrophils, mean platelet volume, high-sensitivity C-reactive protein, and neutrophil-lymphocyte and platelet-lymphocyte ratios were markedly increased when myocardial injury occurred after percutaneous coronary intervention in stable ischemic heart patients. Patients without myocardial injury after percutaneous coronary intervention did not present significant changes in these markers

As shown in figure 1, patients who developed myocardial injury after elective PCI exhibited significantly higher white blood cell (WBC), eosinophil, and neutrophil counts, as well as MPV, hs-CRP, NLR, and PLR values. In contrast, patients without myocardial injury did not show any significant changes in the levels of these biomarkers after PCI.

### Correlation of degree of myocardial injury with inflammatory response

Correlation analysis was used to determine the strength and direction of the association between increased hs-cTnI and IBM levels. hs-CRP levels showed the strongest correlation with myocardial injury (r=0.570, p<0.001). Other biomarkers showed significant but weak correlations. The analysis showed that hs-CRP level after PCI was correlated the most with myocardial injury (ρ 0.579, p<0.001) followed by NLR (ρ 0.190, p<0.001), MPV (ρ 0.182, p<0.001), PLR (ρ 0.180, p<0.001), WBC (ρ 0.166, p<0.001), neutrophil (ρ 0.145, p<0.001), and eosinophil (ρ 0.142, p<0.001) counts; lymphocyte count showed a negative correlation (ρ −0.130, p<0.001), while monocyte and platelet counts after PCI did not correlate with myocardial injury.

Although the correlation of peak post-PCI IBM levels with myocardial injury was significant, the magnitude of the changes was below the clinical decision thresholds; overall, these changes were still within normal limits.

## DISCUSSION

This study demonstrated that PCI in patients with stable CCS induced a systemic inflammatory response that correlated with myocardial injury. Overall, patients with myocardial injury after PCI had higher IBM levels, even though these were within reference values. hs-CRP had the strongest correlation with elective PCI-related myocardial injury, suggesting its potential role in identifying patients at risk of post-PCI complications.

We found that patients with CCS and normal baseline hs-cTnI levels who developed post-PCI myocardial injury were older, had higher baseline blood pressure and LDL levels, had a history of MI and revascularization, used fewer beta-blockers, and were more likely to undergo treatment for left anterior descending artery. There were no differences in the previous use of antiplatelet agents and statins.

The prognostic significance of post-PCI biomarker changes, particularly in patients with stable disease, remains unclear. This study did not aim to evaluate the predictive importance of the biomarker changes related to PCI among CCS patients. Therefore, further studies are required to address this.

Previous reports on the predictive significance of Tn levels after elective PCI are controversial. Previous studies have suggested that baseline Tn levels are more predictive of outcomes than post-PCI Tn elevation. Moreover, procedural myocardial damage may reflect the underlying coronary pathology rather than directly influencing prognosis. Patients presenting with acute coronary syndrome have a worse prognosis after PCI than those with CCS. Studies have also proposed that the prognostic ability of post-PCI Tn is contingent on the clinical appearance of acute coronary syndrome and baseline Tn levels. Recent evidence suggests that a post-PCI Tn increase predicts a 12-month adverse outcome in patients with acute coronary syndrome but not in those with stable conditions. In addition, late outcomes after PCI are mostly linked to preprocedural Tn values and not to biomarker responses. Moreover, if preprocedural Tn levels increase, it may be difficult to differentiate biomarker elevations due to PCI from those due to preprocedural myocardial alterations, and thus it is reasonable to assume that there is an overlap between the prognostic value of baseline and post-PCI biomarker elevations.^(14)^

It has been reported that 30-day and one-year outcomes after elective PCI are related to increased preprocedural Tn values.^(15)^ Thus, the altered inflammatory biomarkers observed in the present study may have a prognostic role in this setting.

Whether periprocedural myocardial damage reflects the causal factors of clinical outcomes or is just a reflection of a more severe coronary disease is yet to be determined and deserves additional clinical research. For instance, an increase in Tn after PCI may be a biomarker for various aspects of aggravated coronary atherosclerosis, such as severe plaque burden,^(16)^ presence of vulnerable plaques, endothelial dysfunction, inflammation, and microvascular injury.^(17)^ Prior findings have identified branch arterial occlusion, dissection, and microvascular hypoperfusion due to thrombus embolization as the main mechanisms of ischemic injury after PCI.^(18)^

This study validates other reports that in the modern practice of PCI in CCS, the occurrence of complicated or failed procedures is uncommon, mainly in low-risk patients. In this study, a new Q-wave MI was observed in <1% of the patients, and the rate of occurrence of angiographic no-reflow was 1.5%. When PCI-related obstacles occur, the complications per se, but not postprocedural Tn, may predict the subsequent prognosis.

The results of this study corroborate the findings of previous studies that detected an increase in the biomarkers of myocardial injury after PCI.^(19)^ Possible explanations include procedure-related microembolization of the atherothrombotic elements or lateral tributary artery occlusion. The management of restenotic lesions-well known to have more fibrotic and less lipid material than natural atheromas-suggests further evidence for the involvement of microembolization of atherothrombotic content in the initiation of myocardial injury and an increase in circulating biomarkers post-PCI. In addition, several other factors, such as lack of statins,^(20)^ complex lesions, bifurcation lesions, pre-dilatation, and total stented length, are associated with elevated Tn levels after PCI.^(21)^

Furthermore, there is evidence that increase in IBM levels, such as hs-CRP, after coronary stenting is related to clinical restenosis.^(22)^ Patients with higher circulating hs-CRP levels after PCI likely have a higher vascular inflammatory status.^(23,24)^ Therefore, the modulation of periprocedural inflammation is appealing to avoid this type of complication. It is important to note that we assessed hematological indices to identify an active inflammatory process and not to provide mechanistic or prognostic insights.

In addition, we noted that the lymphocyte count decreased after PCI. Lymphocytes modulate the inflammatory responses at all levels of atherosclerosis, and lymphopenia has been associated with atherosclerosis progression and poor prognosis in conditions in which inflammation plays a key pathophysiological role.^(25)^

### Clinical relevance

To the best of our knowledge, no other clinical research has demonstrated such clear alterations in proinflammatory biomarkers in cohorts in which acute MI (type 4a) was excluded after successful stent placement in patients with stable disease. The novelty of this study lies in the simultaneous evaluation of both inflammatory and myocardial biomarkers in patients with low-risk stable coronary syndromes. In previous reports, these biomarkers have been continuously assessed distinctly and in high-risk patients.

Our study raises the hypothesis that the degree of the inflammatory response to coronary stenting, as assessed by changes in IBM levels, may be involved in the mechanisms implicated in cardiac injury and its secondary consequences. Since the biomarkers were determined immediately before and after PCI, we concluded that other factors, such as medications used, LDL, and blood pressure levels, were not responsible for these changes.

### Study limitations

The present study had several limitations. First, it is an observational exploration, and as such, has limitations intrinsic to this design. Second, baseline Tn levels can be associated with cardiovascular risk factors, more extensive CCS, and comorbidities, all of which tend to occur among patients with altered baseline levels of IBM. The inclusion of low-risk patients in the current study might have had consequences such as a reduced incidence of PCI-related complications. Third, the small sample size decreased the statistical power of the angiographic and clinical outcomes. Further, any insights into the clinical outcomes were restricted because no systematic angiographic follow-up was performed.

## CONCLUSION

This investigation demonstrated that elective percutaneous coronary intervention in patients with stable disease induces a systemic inflammatory response, which correlates with the burden of myocardial injury. Further studies are needed to investigate the prognostic implications of these findings and explore strategies for modulating periprocedural inflammation. Clinical trials using anti-inflammatory drugs, such as low-dose colchicine, during the periprocedural period of percutaneous coronary intervention may help understand the role of inflammation in myocardial injury after percutaneous coronary intervention.
